# Labor epidural analgesia versus without labor epidural analgesia for multiparous women: a retrospective case control study

**DOI:** 10.1186/s12871-021-01355-0

**Published:** 2021-04-28

**Authors:** Shuzhi Luo, Zhaowen Chen, Xujian Wang, Changyu Zhu, Shili Su

**Affiliations:** 1 Department of Anesthesiology, Shandong Province Maternal and Child Health Care Hospital, 238 East Road of Jingshi, Jinan, Shandong P.R. China; 2 Department of Gynaecology and Obstetrics, Shandong Province Maternal and Child Health Care Hospital, 238 East Road of Jingshi, Jinan, Shandong P.R. China; 3 Key Laboratory of Birth Regulation and Control Technology of National Health Commission of China, 238 East Road of Jingshi, Jinan, Shandong P.R. China

**Keywords:** Epidural, Analgesia, Labor duration, Labor stage, Multipara, Maternal and neonatal outcomes

## Abstract

**Background:**

Labor epidural analgesia (LEA) effectively relieves the labor pain, but it is still not available consistently for multiparous women in many institutions because of their obviously shortened labor length.

**Methods:**

A total of 811 multiprous women were retrospective enrolled and firstly divided into two groups: LEA group or non-LEA group. And then they were divided into seven subgroups and analyzed according to the use of LEA and cervical dilation. The primary outcomes (time intervals, blood loss and Apgar scores) and secondary outcomes (maternal demographic characteristics and birth weight) were collected by checking electronic medical records.

**Results:**

The prevalence of using LEA in multiprous women was 54.5 %. Using LEA significantly lengthened the duration of labor stage by 56 min (*P* < 0.001), increased the blood loss (*P* < 0.001) and lowered Apgar scores (*P* = 0.001). In the comparison of sub-group analysis, using LEA can obviously prolong the duration of first-second stage in women with 2 cm cervical dilation (*P* < 0.001) and 3 cm cervical dilation (*P* = 0.014), while there was no significant difference with 4 cm or more cervical dilation (*P* = 0.69). Using LEA can significantly increased the blood loss when the initiation of LEA in the women with 2 cm cervical dilation (*P* < 0.001) and 3 cm cervical dilation (*P* = 0.035), meanwhile there were no significantly differences in the women with 4 cm or more cervical dilation (*P* = 0.524). Using LEA can significantly lower the Apgar scores when the initiation of LEA in the women with 2 cm cervical dilation (*P* = 0.001) and 4 cm or more cervical dilation (*P* = 0.025), while there were no significantly differences in the women with 3 cm cervical dilation (*P* = 0.839).

**Conclusions:**

Labor epidural analgesia for the multiparous woman may alter progress of labor, increase postpartum blood loss and lower Apgar scores. Early or late initiation of LEA should be defined as with cervical dilatation of less or more than 3 cm and the different effect should be understand.

**Trial registration:**

ChiCTR2100042746. Registered 27 January 2021-Prospectively registered, http://www.chictr.org.cn.

## Background

Epidural analgesia effectively relieves the pain during the progress of labor, but it is still not available consistently in many institutions. Though the rate of epidural analgesia using has been increased in China, nearly 57 % in some institutions recently [1], only 4 % of total women and 21.6 % of low-risk women received labor epidural analgesia in the whole world [2, 3].

Epidural analgesia is believed to increase the vaginal delivery rate, enhance safety, outcomes and satisfaction[1, 4], many factors from the women and obstetricians still influence the use of epidural analgesia. A recently retrospective cohort study reported that women who received epidural analgesia administration may have adverse effects on the labor process, which may increase the morbidity risk for the mother [5]. Some maternal-fetal adverse outcomes which the use of labor epidural analgesia may lead to, such as prolonged labor duration, back pain, postpartum hemorrhage and lower Apgar scores [6–12], prevented it from widely used. The obstetricians often concern that epidural analgesia may alter the progress of labor to prolong the duration of stage, especially because of weakened and delayed pushing due to LEA in the second stage. They also thought that women without epidural analgesia can make immediate, long, sustained pushes with each contraction to assist vaginal birth to shorten the duration of labor stage, though 85 % midwives allowed women using epidural analgesia with delayed pushing [13] and delayed pushing did result in a longer second stage [6–8, 14]. Back pain after labor epidural analgesia is often concerned by women, though it has been confirmed that the labor pain relief technique did not trigger the increased risk of back pain [9]. Even so, it is thought that in the absence of a medical contraindication, maternal request was a sufficient medical indication for pain relief during labor [15].

The multiparous women seldom scheduled for delivery to admit to the hospital in advance and usually went to hospital for delivery after labor onset. Meanwhile, the length of labor in multiparous women was obviously shortened and was a mean of 6 h without regional analgesia. As a result, they always admitted with cervical dilatation of 2 to 3 cm or more, even the delivery was right now. Therefore, it is difficult to decide to use LEA because of progressive cervical dilatation and the highly variable length of labor stage. Though various forms of alternative pain relief were given to women, it was hard to assess the outcomes clearly. We thought that the labor epidural analgesia for multiparous women should be deeply concerned and conducted this study to evaluate the use of labor epidural analgesia for multiparous women. In this study, we just evaluated multiparous women with singleton pregnancy and vaginal delivery, excluded vaginal birth after cesarean section and cesarean delivery.

## Methods

### Ethics and study design

This study was approved by the Ethics Committee of Shandong Province Maternal and Child Health Care Hospital (reference number: 2020SYY006). The study was also registered at www.chictr.org.cn (registration number: ChiCTR2100042746) as a retrospective case-control study.

This study was performed at Shandong Province Maternal and Child Health Care Hospital and collected the cases between 1 January, 2019 and 30 June, 2019. The inclusion criteria were multiparous women, singleton pregnancy, vaginal delivery and gestational age of 37 weeks. Exclusion criteria were pregnancy complications needing additional interventions, vaginal birth after cesarean section and cesarean delivery.

The characteristics of LEA were as follows: after catheterization of epidural catheter was perfectly performed, 3 ml of 1.5 % lidocaine was administered as the test dose. Then, labor analgesia was initiated with 8–12 ml of 0.075 % ropivacaine with 0.5 µg/ml of sufentanil. All the women were provided continuous epidural infusion (CEI) which was at a constant rate of 10 ml/h and patient-controlled epidural analgesia (PCEA) which was bolus of 5 ml with a 30-minute lockout using the analgesia pumps. The labor analgesia was performed until 2 h after delivery.

Because labor accelerated much faster in multiparas and the first- or second- stage of labor was very difficult to demarcated, first- and second- stage of labor for multiparas were usually put together to record as first-second stage in our institute. Blood loss estimation was quantified by regularly weighing the plastic bag which was placed under the pelvis of the women for blood collection.

The blood collection began immediately after fetal birth and ended with no unusual bleeding. During this time, the placentas were delivery and lacerations were repaired. Once the postpartum hemorrhage occurred, treatment should be performed to control postpartum hemorrhage.

The Apgar scores was scored at 1 and 5 min by the attending midwife or present obstetrician and subsequently recorded in the database.

The primary outcomes were time intervals (duration between initial vaginal examination after labor onset and delivery in women without LEA, duration between initiation of the labor analgesia and delivery in women with LEA, duration of first-second stage), duration ratio (the proportion of the duration between initial vaginal examination or LEA and delivery to the duration of first-second stage), blood loss and Apgar scores. Secondary outcomes were collected such as maternal demographic characteristics (age, height, weight, BMI = body mass index, gestational weight gain and gestational age), duration of third stage and birth weight.

We sought and checked electronic medical records of multiprous women who under vaginal delivery and women with incomplete records were excluded. Then we found out the time of initial vaginal examination and cervical dilation after onset of labor, and got the duration between initial vaginal examination and delivery for the women without labor epidural analgesia. In the women with labor epidural analgesia, we checked the time of initiation of labor analgesia and cervical dilation, then we worked out the duration between initiation of labor analgesia and delivery.

### Statistical analysis

The statistical analysis of the data was conducted using SPSS, version 26.0. The data were tested by the Shapiro-Wilk test for normal distribution firstly. The normal distributed data were presented as mean ± standard deviation (SD) and analyzed using t-test or Chi-squared Test (χ^2^-test). The non-normal distributed data were presented as medians and quartiles and analyzed with the non-parametric test. Differences with *P* < 0.05 were considered significantly.

## Results

**Fig. 1:**
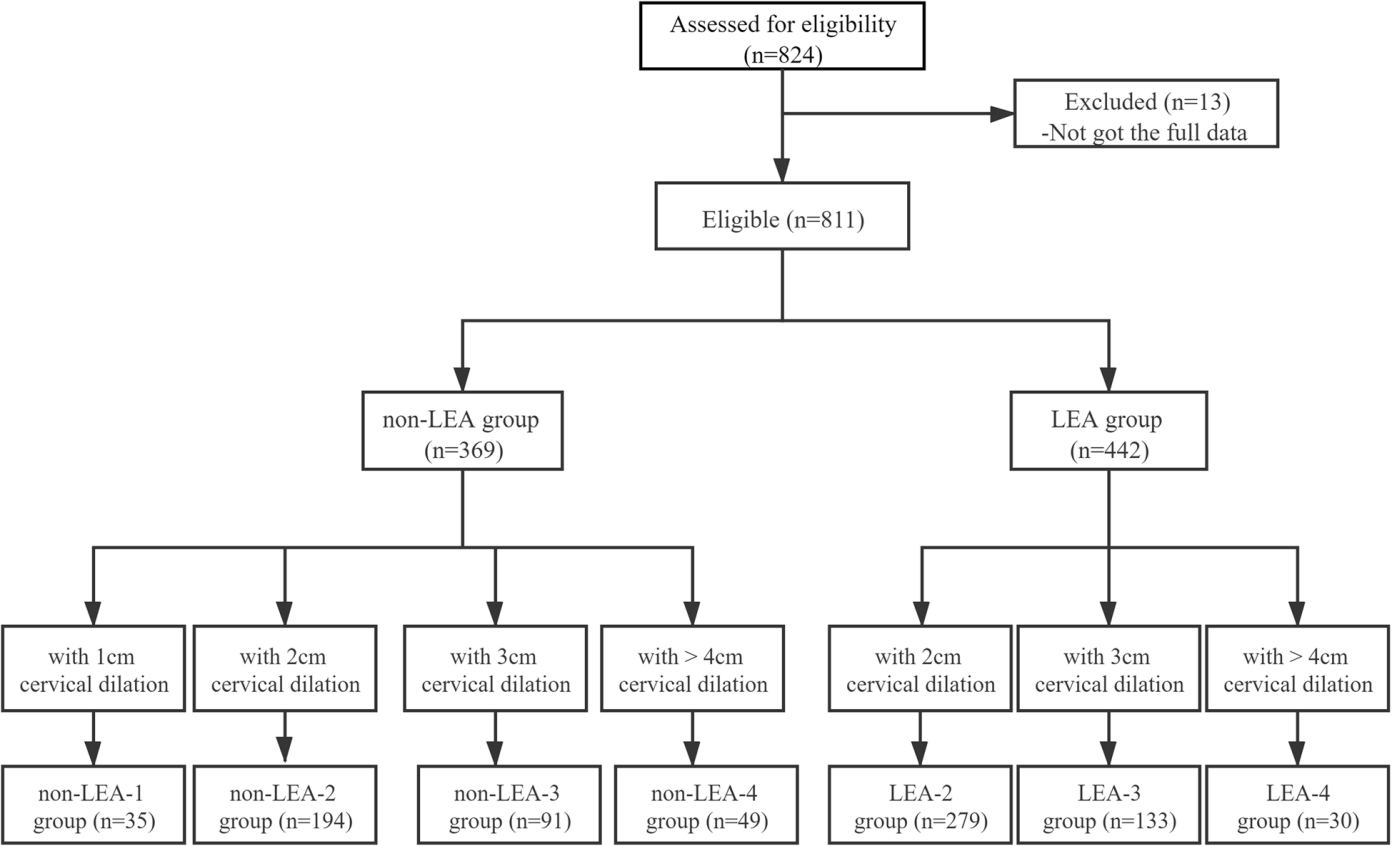
Flow diagram of study.

A total of 824 women were assessed for eligibility in the study. All the cases were reviewed and 13 women were excluded from this study because of incomplete information. 811 women were enrolled to be analyzed. There were 369 women in the birth process without labor epidural analgesia (non-LEA group) and 442 women using labor epidural analgesia (LEA group). The prevalence of multiprous women receiving labor epidural analgesia was 54.5 % (422/811). According to cervical dilation, 369 women without labor epidural analgesia were divided into 4 groups: the women with 1 cm cervical dilation (non-LEA-1) group, the women with 2 cm cervical dilation (non-LEA-2) group, the women with 3 cm cervical dilation (non-LEA-3) group, the women with cervical dilation more than 4 cm (non-LEA-4) group, and 442 women with labor epidural analgesia were divided into 3 groups: the women with 2 cm cervical dilation (LEA-2) group, the women with 3 cm cervical dilation (LEA-3) group, the women with cervical dilation more than 4 cm (LEA-4) group (Fig. 1).

### Comparison of non-LEA group and LEA group

The data for the total of 811 women were shown in Table 1. There were no significantly differences with maternal age, height, weight, gestational weeks, duration of third stage and birth weight between non-LEA group and LEA group. There were significantly differences between the two groups in weight (*P* = 0.022), BMI (*P* = 0.022) and gestational age (*P* = 0.011). The duration between initial vaginal examination and delivery in non-LEA group was longer than the duration between initiation of epidural analgesia and delivery in LEA group [133 (86.5–222) vs. 120 (74-181.25), Z/T = -3.358, *P* = 0.001]. The duration of the first-second stage in non-LEA group was shorter in LEA group [296 (234.5–409) vs. 352.5 (265-443.75), Z/T = -4.06, *P* < 0.001]. Epidural analgesia was found to lengthen the duration of first-second stage by 56 min. Though there was no significantly difference in the prevalence of postpartum hemorrhage between non-LEA group and LEA group (2.17 % vs. 1.81 %, *P* = 0.715), the blood loss of non-LEA group was significantly less than LEA group (*P* < 0.001). There was no Apgar scores less than seven at one minute in both two groups, but the Apgar scores of non-LEA group was significantly higher than LEA group (*P* = 0.001).

**Table 1:** Characteristics and outcomes of the total non-LEA group and LEA group.

	non-LEA group (*n* = 369)	LEA group (*n* = 442)	Z/T	*P*-value
Age (years)	32 (30–34)	32 (30–34)	-0.666	0.506
Height (cm)	162 (160–165)	163 (160–165)	-0.789	0.43
Weight (kg)	73 (67–79)	74 (68-80.63)	-2.296	0.022
BMI (kg/m2)	27.55 (25.62–29.67)	28.12 (25.85–30.38)	-2.148	0.032
Gestational weight gain (kg)	15 (12-17.5)	15 (12–18)	-0.006	0.995
Gestational age (weeks)	39.4 (38.6–40.1)	39.5 (39.1-40.23)	-2.546	0.011
Duration between initial vaginal examination or epidural analgesia and delivery (mins)	133 (86.5–222)	120 (74-181.25)	-3.358	0.001
Duration of first-second stage (mins)	296 (234.5–409)	352.5 (265-443.75)	-4.06	< 0.001
Duration of third stage (mins)	5 (5–7)	5 (5–7)	-1.405	0.16
Blood loss (ml)	240 (220–280)	260 (230–290)	-3.941	< 0.001
Postpartum hemorrhage (n)	2.17 % (8)	1.81 % (8)	0.133	0.715
Apgar score	10 (10–10)	10 (10–10)	-3.318	0.001
Birth weight (g)	3045.81 ± 404.36	3453.89 ± 404.85	-1.685	0.092

### Comparison of non-LEA group with 1 and 2 cm cervical dilation

**Fig. 2:**
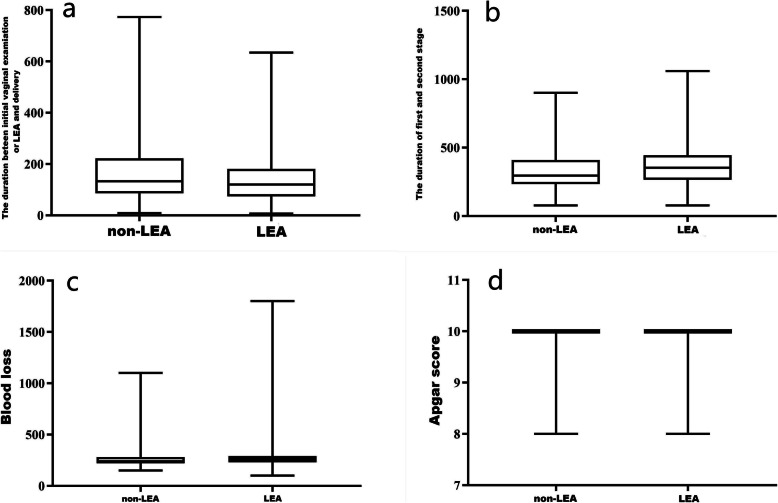
**a** Using LEA can significantly shorten the duration between cervical dilation of 2 cm and delivery compared to non-LEA-2 group (*P* < 0.001) and prolong the duration of the women with with cervical dilation more than 4 cm compared to non-LEA-4 group (*P* = 0.043), while there was no significant difference between non-LEA-3 group and LEA-3 group (*P* = 0.767). **b** Using LEA can obviously prolong the duration of first-second stage in the women with 2 cm cervical dilation (*P* < 0.001) and 3 cm cervical dilation (*P* = 0.014), while there was no significant difference in the two groups with cervical dilation more than 4 cm (*P* = 0.69). **c** This picture shows that the timing of epidural placement at 3 cm cervical dilation is a turning point. **d** Once the cervical dilation is more than 2 cm, no matter when is the LEA applied, the postpartum blood loss of LEA group is more than the non-LEA group.

Comparison of characteristics and outcomes between the women with 1 and 2 cm cervical dilation in non-LEA group was shown in Table 2. There were no significantly differences with height, weight, BMI, gestational weight gain, gestational age, duration of third stage, blood loss, Apgar scores and birth weight between non-LEA-1 group and non-LEA-2 group. There was significantly difference in maternal age between the two groups (*P* = 0.037). The duration between initial vaginal examination and delivery of non-LEA-1 group was significantly longer than non-LEA-2 group [217 (183–321) vs. 164.5 (101.75–243.5), Z/T = -3.344, *P* = 0.001], but there was no significantly difference with duration of first-second stage in the two groups. The duration ratio of non-LEA-1 group was also obviously higher than non-LEA-2 group (Fig. 2a-c).

**Table 2:** Characteristics and outcomes of the women with 1 and 2 cm cervical dilation in non-LEA group.

	non-LEA-1 group (*n* = 35)	non-LEA-2 group (*n* = 194)	Z/T	*P*-value
Age (years)	34 (31–36)	32 (30–34)	-2.084	0.037
Height (cm)	160 (160–163)	162 (160–165)	-1.475	0.14
Weight (kg)	72 (65.5–79)	72.5 (66–79)	-0.517	0.605
BMI (kg/m2)	27.34 (25.71–29.38)	27.61 (25.39–30.08)	-0.251	0.802
Gestational weight gain (kg)	14 (10–16)	15 (11.88–17.63)	-1.16	0.246
Gestational age (weeks)	39.1 (38.1–40)	39.4 (38.6–40.1)	-1.315	0.188
Duration between initial vaginal examination and delivery (mins)	217 (183–321)	164.5 (101.75–243.5)	-3.344	0.001
Duration of first-second stage (mins)	373 (272–471)	312.5 (231.5–408)	-1.9	0.057
Duration ratio	0.69 (0.52–0.91)	0.59 (0.38–0.77)	-2.118	0.034
Duration of third stage (mins)	5 (4–7)	5 (5–7)	-0.051	0.96
Blood loss (ml)	260 (230–300)	240 (220–280)	-1.774	0.076
Apgar score	10 (10–10)	10 (10–10)	-0.531	0.595
Birth weight (g)	3288 ± 404.54	3394.12 ± 416.98	-1.392	0.165

### Comparison of non-LEA-2 group and LEA-2 group

The data for the women with 2 cm cervical dilation in non-LEA-2 group and LEA-2 group were shown in Table 3. There were no significantly differences with maternal age, height, BMI and duration of third stage. There were significantly differences between non-LEA-2 group and LEA-2 group in weight (*P* = 0.027), gestational age (*P* = 0.031) and birth weight (*P* = 0.028). Duration from cervical dilation of 2 cm to delivery of non-LEA-2 group was significantly longer than LEA-2 group by 40 min [164.5 (101.75–243.5) vs. 128 (78–196), Z/T = -4.361, *P* < 0.001], but the duration of first-second stage of non-LEA-2 group was obviously shorter than LEA-2 group by 50 min [312.5 (231.5–408) vs. 362 (273–452), Z/T = -3.534, *P* < 0.001]. The duration ratio of non-LEA-2 group was significantly higher than LEA-2 group [0.59 (0.38 ~ 0.77) vs. 0.36 (0.23 ~ 0.54), Z/T = -8.055, *P* < 0.001). The blood loss of non-LEA-2 group was less than LEA-2 group (*P* < 0.001) and the Apgar scores of non-LEA-2 group was higher than LEA-2 group (*P* = 0.001).

**Table 3:** Characteristics and outcomes of the women with 2 cm cervical dilation in non-LEA group and LEA group.

	non-LEA-2 group (*n* = 194)	LEA-2 group (*n* = 279)	Z/T	P-value
Age (years)	32 (30–34)	32 (30–34)	-0.115	0.909
Height (cm)	162 (160–165)	163 (160–165)	-1.08	0.28
Weight (kg)	72.5 (66–79)	74 (69–81)	-2.207	0.027
BMI (kg/m2)	27.61 (25.39–30.08)	28.2 (25.78–30.47)	-1.778	0.075
Gestational weight gain (kg)	15 (11.88–17.63)	15 (12-18.5)	-0.309	0.757
Gestational age (weeks)	39.4 (38.6–40.1)	39.5 (39-40.3)	-2.152	0.031
Duration from cervical dilation of 2 cm to delivery (mins)	164.5 (101.75–243.5)	128 (78–196)	-4.361	< 0.001
Duration of first-second stage (mins)	312.5 (231.5–408)	362 (273–452)	-3.534	< 0.001
Duration ratio	0.59 (0.38–0.77)	0.36 (0.23–0.54)	-8.055	< 0.001
Duration of third stage (mins)	5 (5–7)	5 (5–6)	-0.595	0.552
Blood loss (ml)	240 (220–280)	260 (240–290)	-3.657	< 0.001
Apgar score	10 (10–10)	10 (10–10)	-3.226	0.001
Birth weight (g)	3394.12 ± 416.98	3479.71 ± 413.46	-2.207	0.028

### Comparison of non-LEA-3 group and LEA-3 group

The data for the women with 3 cm cervical dilation in non-LEA-3 group and LEA-3 group were exhibited in Table 4. There were no significantly differences with maternal age, height, weight, BMI, gestational weight gain, gestational age, duration ratio, duration of third stage and birth weight. There were no significantly differences with the duration from cervical dilation of 3 cm to delivery of non-LEA-3 group and LEA-3 group [111 (78–148) vs. 107 (66.5-154.5), Z/T = -0.296, *P* = 0.767], but the duration of first-second stage of non-LEA-3 group was shorter than LEA-3 group by 50 min [283 (232–388) vs. 335 (260-432.5), Z/T = -2.451, *P* = 0.014]. The blood loss of non-LEA-3 group was less than LEA-3 group (*P* = 0.035). There was no significantly difference with the Apgar scores between the two groups (*P* = 0.839).

**Table 4:** Characteristics and outcomes of the women with 3 cm cervical dilation in non-LEA group and LEA group.

	non-LEA-3 group (*n* = 91)	LEA-3 group (*n* = 133)	Z/T	*P*-value
Age (years)	32 (30–34)	31 (29–34)	-0.692	0.489
Height (cm)	163 (160–166)	163 (160–166)	-0.536	0.592
Weight (kg)	74 (67.5–78)	75 (67-80.5)	-0.728	0.467
BMI (kg/m2)	27.54 (25.77–29.24)	28.23 (26.04–30.12)	-1.407	0.16
Gestational weight gain (kg)	15 (13–18)	15 (12–18)	-0.591	0.554
Gestational age (weeks)	39.4 (39-40.2)	39.6 (39.1–40.3)	-1.27	0.204
Duration from cervical dilation of 3 cm to delivery (mins)	111 (78–148)	107 (66.5-154.5)	-0.296	0.767
Duration of first-second stage (mins)	283 (232–388)	335 (260-432.5)	-2.451	0.014
Duration ratio	0.4 (0.25–0.5)	0.32 (0.23–0.46)	-1.914	0.056
Duration of third stage (mins)	5 (5–7)	6 (5–7)	-1.135	0.256
Blood loss (ml)	240 (220–280)	260 (230–280)	-2.104	0.035
Apgar score	10 (10–10)	10 (10–10)	-0.204	0.839
Birth weight (g)	3457.42 ± 345.77	3412.11 ± 397.63	0.882	0.379

### Comparison of non-LEA-4 group and LEA-4 group

The data for the women with cervical dilation more than 4 cm in non-LEA-4 group and LEA-4 group were shown in Table 5. There were no significantly differences with maternal age, height, weight, BMI, gestational weight gain, gestational age, duration ratio, duration of third stage and birth weight. Duration from cervical dilation > 4 cm to delivery of non-LEA-4 group was shorter than LEA-4 group by 30 min [81 (32–103) vs. 101.5 (55.75-193.75), Z/T = -2.026, *P* = 0.043]. There were no significantly differences with the duration of first-second stage between the two groups [281 (226–408) vs. 282.5 (240.75–435.5), Z/T = -0.399, *P* = 0.69]. The blood loss of the two groups was no significantly differences (*P* = 0.524). Time intervals, duration ratio and blood loss are shown in Fig. 2. The Apgar scores of non-LEA-4 group was higher than LEA-4 group (*P* = 0.025).

**Table 5:** Characteristics and outcomes of the women with cervical dilation more than 4 cm in non-LEA group and LEA group.

	non-LEA-4 group (*n* = 49)	LEA-4 group (*n* = 30)	Z/T	*P*-value
Age (years)	32 (29–34)	32 (30-33.25)	-0.03	0.976
Height (cm)	163 (160–166)	160 (159.75-165.25)	-0.523	0.601
Weight (kg)	72 (68–79)	72 (66.38–77.65)	-0.399	0.69
BMI (kg/m2)	27.73 (25.53–29.38)	27.01 (25.68–29.3)	-0.551	0.582
Gestational weight gain (kg)	15 (12.5–18)	12.8 (10.08-16)	-1.28	0.201
Gestational age (weeks)	39.5 (39-40.1)	39.2 (38.9–39.5)	-1.321	0.187
Duration from cervical dilation > 4 cm to delivery (mins)	81 (32–103)	101.5 (55.75-193.75)	-2.026	0.043
Duration of first-second stage (mins)	281 (226–408)	282.5 (240.75–435.5)	-0.399	0.69
Duration ratio	0.24 (0.12–0.37)	0.34 (0.14–0.5)	-1.829	0.067
Duration of third stage (mins)	5 (5-6.5)	6 (5-7.25)	-1.397	0.163
Blood loss (ml)	240 (220–280)	260 (220–280)	-0.637	0.524
Apgar score	10 (10–10)	10 (10–10)	-2.242	0.025
Birth weight (g)	3440.41 ± 444.35	3399 ± 341.23	0.437	0.663

## Discussions

**Fig. 3:**
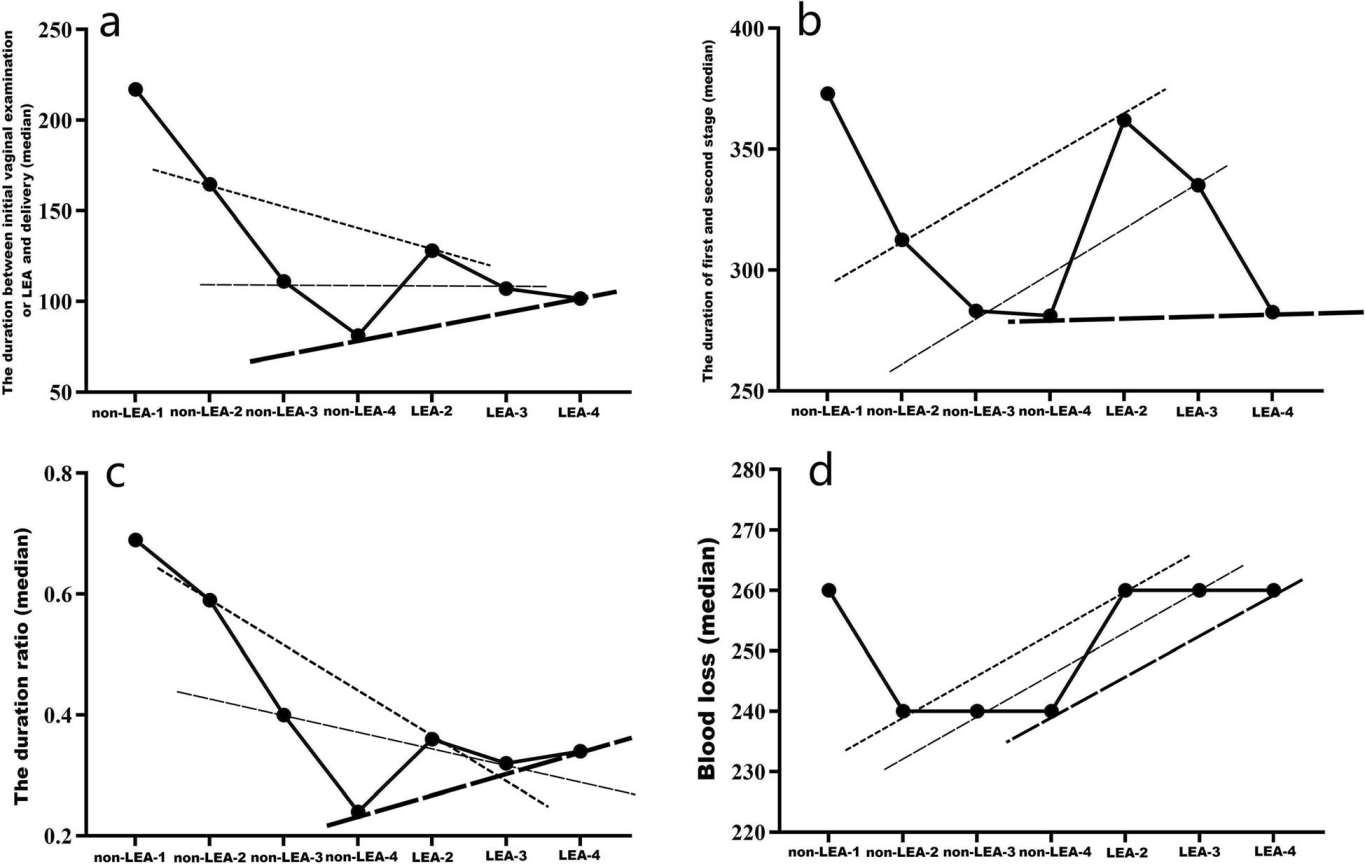
**a** Comparison of duration between initial vaginal examination or LEA and delivery in the two groups. The duration of non-LEA group was significantly longer than LEA group (*P* = 0.001). **b** The duration of the first-second stage in non-LEA group was shorter in LEA group (*P* < 0.001). **c** The blood loss of non-LEA group was significantly less than LEA group (*P* < 0.001). **d** The Apgar score of non-LEA group was significantly higher than LEA group (*P* = 0.001).

In this present study, we found that using LEA did lengthen the stage of labor compared to the non-LEA group (*P* < 0.001) and nearly prolonged the length of stage by 56 min (Table 1; Fig. 3). It is similar to the use of LEA in nulliparous women though it will be much longer. The prolonged time was particularly noticeable in multiparous women because of the shorter labor duration compared to nulliparous women. This results also proved that intervention during the second stage of labor should be made based on more than 2 h for both nulliparous and multiparous women [11].

Because of particularity of cervical dilation in multiparous women, the measurement of dilation was very difficult, especially from 2 to 3 cm cervical dilation. The accuracy of cervical dilation was verified firstly through the comparison between non-LEA-1 group and non-LEA-2 group. The results provided abundant evidence that the cervical dilation of the total women should be recognized (Table 2; Fig. 2a-c). Then we compared the groups divided according to the use of LEA and cervical dilation.

We found that when LEA was used too early or too late, there was a exactly opposite effect on the duration between initiation of epidural analgesia and delivery though a randomized controlled trial study reported a low concentration of epidural local anesthetic does not affect the duration of the second stage of labor [16]. Early initiation of epidural analgesia significantly shortened the duration of analgesia perhaps because labor epidural analgesia induced early cervical dilation and descent of the fetal head [17]. The obviously prolonged duration of first-second stage due to early initiation of epidural analgesia was like to the growing consensus of opinion on that labor epidural analgesia did prolonged duration of the second stage [18]. On the contrary, late initiation of epidural analgesia prolonged the duration of analgesia and did not alter the duration of first-second stage (Fig. 2a). When timing of epidural placement was at 3 cm cervical dilation, the duration of epidural analgesia was not prolonged but the duration of first-second stage was still lengthened. Early initiation of epidural analgesia accelerated the process of cervical dilation while prolonged the total labor stage and late initiation did not prolonged the total labor stage but the women experienced longer analgesia (Fig. 2a-b). Through the duration ratio, we found that 3 cm cervical dilation was a turning point (Fig. 2c). The obstetricians were usually worried that epidural analgesia was used too late for the multiparous women because of the highly variable duration of stage, especially when the cervical dilation was more than 4 cm. In fact, even women were with cervical dilation more than 4 cm, they would experience labor pain nearly 80 min (Table 5). Though using LEA prolonged the duration of analgesia when initiation of epidural analgesia at cervical dilation more than 4 cm, it did not lengthen the duration of first-second stage. According to this, we suggested that unless the delivery was right now, the multiparous women should be applied labor epidural analgesia no matter how the cervical dilation if the women wanted labor epidural analgesia.

In our study, we found that the blood loss was increased significantly in the LEA groups until the initiation of labor epidural analgesia for the women with cervical dilation more than 4 cm (Fig. 2d), though there was no significantly difference in the prevalence of postpartum hemorrhage between non-LEA group and LEA group (Table 1; Figs. 3 and 2d). Labor epidural anesthesia should be discussed as a risk factor for postpartum hemorrhage though epidural anesthesia had a protective effect on women with postpartum hemorrhage regardless of the effect of the epidural on the occurrence of postpartum hemorrhage [10]. Our results suggest that once epidural analgesia was applied, no matter the presence of prolonged stage of labor, greater blood loss or postpartum hemorrhage should be vigilant. The increased blood loss without immediate and correct management often led to severe postpartum hemorrhage.

We found that early initiation and late initiation of epidural analgesia both led to lower Apgar scores though there was no Apgar scores less than seven at one minute while there was no significantly difference when the timing of epidural placement was at 3 cm cervical dilation (Tables 1, 3 and 5; Fig. 3). Though a latest Cochrane reviewed comprising 40 trials found that labor epidural analgesia have not an immediate effect on neonatal status as determined by Apgar scores or in admissions to neonatal intensive care [17], the use of epidural analgesia was associated with higher odds of low Apgar scores and admission to the neonatal intensive care unit [3, 12]. Because those studies were only for nulliparous women, there is a need to draw critical attention to evaluate the use of labor epidural analgesia in multiparous women with impacts on neonatal outcomes. Our results suggested that when multiparous women were applied LEA, the obstetricians and midwives should pay more attention during labor and delivery.

According to our results, though using labor epidural analgesia at 3 cm cervical dilation has the least effect on multiparous woman, early or late initiation of epidural analgesia just have few negative effects on time intervals, blood loss and Apgar score and there were no serious adverse outcomes. Labor epidural analgesia can safely be used at any stage of labor, including the second stage and the time to initiate epidural analgesia is dependent upon women’s requests [19, 20].

## Conclusions

In conclusion, labor epidural analgesia for the multiparous woman may alter the progress of labor, increase the postpartum blood loss and lower the Apgar scores. For multiparous woman, early initiation should be defined as with cervical dilatation of less than 3 cm, and late initiation with cervical dilatation of 3 cm or more. The different effect of early or late initiation of LEA should be understand. Early initiation of epidural analgesia prolongs the total labor stage but accelerates the process of cervical dilation and late initiation dose not alter the total labor stage but leads to longer duration of epidural analgesia. Only early initiation of epidural analgesia increases postpartum blood loss. Both early and late initiation of epidural analgesia equally lower the Apgar scores. Neither early nor late initiation of epidural analgesia does not increases incidence of postpartum hemorrhage and neonatal asphyxia. Obstetricians must be woken up to provide the epidural analgesia for multiparous woman and each woman should be individually assessed and apprised no matter the cervical dilation once the labor was onset. Greater blood loss and lower Apgar scores must be vigilant no matter the timing of epidural placement.

## Data Availability

The datasets used and analysed during the current study are available from the corresponding author on reasonable request.

## Abbreviations

LEA: labor epidural analgesia
non-LEA: without labor epidural analgesia
SD: standard deviation
BMI: body mass index
CEI: continuous epidural infusion
PCEA: patient-controlled epidural analgesia
